# Mechanical Anisotropy of Injection-Molded PP/PS Polymer Blends and Correlation with Morphology

**DOI:** 10.3390/polym15204167

**Published:** 2023-10-20

**Authors:** Tetsuo Takayama, Rin Shibazaki

**Affiliations:** Graduate School of Organic Materials Science, Yamagata University, Yonezawa 992-8510, Japan

**Keywords:** interfacial interaction force, morphology, short-beam shear tests, yield condition, yield initiation stress

## Abstract

The molecular orientation formed by melt-forming processes depends strongly on the flow direction. Quantifying this anisotropy, which is more pronounced in polymer blends, is important for assessing the mechanical properties of thermoplastic molded products. For injection-molded polymer blends, this study used short-beam shear testing to evaluate the mechanical anisotropy as a stress concentration factor, and clarified the correlation between the evaluation results and the phase structure. Furthermore, because only shear yielding occurs with short-beam shear testing, the yielding conditions related to uniaxial tensile loading were identified by comparing the results with those of three-point bending tests. For continuous-phase PP, the phase structure formed a sea-island structure. The yield condition under uniaxial tensile loading was interface debonding. For continuous-phase PS, the phase structure was dispersed and elongated in the flow direction. The addition of styrene–ethylene–butadiene–styrene (SEBS) altered this structure. The yielding condition under uniaxial tensile loading was shear yielding. The aspect ratio of the dispersed phase was found to correlate with the stress concentration factor. When the PP forming the sea-island structure was of continuous phase, the log-complex law was sufficient to explain the shear yield initiation stress without consideration of the interfacial interaction stress.

## 1. Introduction

Thermoplastics are used in widely diverse applications, from minor daily necessities to automobiles, because of their lighter weight and superior moldability than those of metals and ceramics [1,2,3]. Moreover, because thermoplastics have lower melting temperatures than metals or ceramics, they can be melt-molded with the expenditure of low energy costs. Among these molding methods, injection molding is often applied for thermoplastics because it enables near-net-shape molding and because it is excellent for mass production [2,4]. Depending on the required shape of the molded product, extrusion [5] or blow molding [6] may be applied, and depending on the required properties, a polymer blend may be prepared by melt mixing and then melt-molded into a product [7,8,9,10,11,12,13,14,15,16,17]. Examples of polymer blends include polypropylene (PP)/polystyrene (PS) blends [7,8,9], polyethylene terephthalate (PET)/polyethylene [10], and PET/PP blends [11], high-impact polystyrene (PS-HI) blends [12,13] blended with elastomers to improve the toughness of polystyrene, and polycarbonate (PC)/acrylonitrile-butadiene-styrene (ABS) copolymers, which offer a wide range of controllable processing and mechanical properties [14,15,16,17]. Polymer blend is a generic term for materials that are composites of two or more polymers. Polymer blends are prepared to achieve physical properties that cannot be achieved with single polymers. However, it is known that most blends thermodynamically form a phase-separated structure, and this is one of the reasons why it is difficult to obtain the desired physical properties [18]. This is also true for mechanical properties, and, in particular, the mechanical anisotropy of a molded product is strongly dependent on the phase-separated structure that is formed. Therefore, it is very important to quantify the anisotropy when discussing the mechanical properties of molded polymer blends. As for PP/PS, which is the subject of this study, the relationship between its phase structure and mechanical properties has been reported, but descriptions of its mechanical anisotropy could not be found in the authors’ investigation [7,8,9].

Various studies have been conducted to examine the correlation between the phase structure and mechanical anisotropy of polymer blends. For instance, Li et al. performed thin-wall injection molding of a polymer blend of polypropylene (PP) and thermoplastic rubber, and cut specimens from the resulting molded product in the direction parallel (MD) and perpendicular (TD) to the flow direction for tensile testing [19]. They found that elongation at the break of specimens cut in the TD direction was about twice as large as that of specimens cut in the MD direction. The reasons for their finding are discussed in terms of the morphology of the dispersed phase. A correlation between elongation at the break of the polymer blend and the morphology of the dispersed phase has been reported [20]. That study reported that the phase structure of a 20 wt.% blend of PP with PS varies with the melt viscosity of PP and that it can be organized by the viscosity ratio of PS to PP.

Such reports discuss the mechanical properties and the anisotropy of the morphology of the dispersed phase which is formed via injection molding. Nevertheless, quantitative analysis of morphology effects of the dispersed phase on the mechanical properties of polymer blends is difficult, as is clarification of the factors contributing to these effects. For some areas, conventional evaluation methods are inadequate for analyzing the mechanical anisotropy which occurs in polymer blend molded products.

The evaluation of mechanical anisotropy of polymer molded products is mainly performed via the cutout method [19]. The cutout method evaluates mechanical anisotropy by performing mechanical tests on specimens cut in a specific direction and comparing the results between the cutout directions. This method is effective for evaluating the mechanical anisotropy of two-dimensional flat surfaces such as films, but is not suitable for evaluating the mechanical anisotropy of three-dimensional objects such as injection-molded products. Residual stresses in the molded product may relax when the injection-molded product is machined, leaving the possibility that the cut-out method may not correctly evaluate the mechanical anisotropy of the molded product [21]. Another method for evaluating mechanical anisotropy is indentation hardness testing [22], with results based on the ratio of indentation lengths obtained from testing. This simple method requires no preparation of specimens, as is necessary for the cut-out method.

Although the reports explained above present methods for quantitatively evaluating mechanical anisotropy, they cannot be characterized as methods for quantitatively analyzing the correlation between a polymer blend’s phase structure and its mechanical properties. Moreover, they do not clarify the factors causing such anisotropy. As demonstrated by the discussion presented above, no study has provided evidence clarifying the mechanisms of mechanical anisotropy in polymer blend injection-molded products.

Against this background, the authors have proposed a method for evaluating the mechanical anisotropy of polymer injection-molded parts in three dimensions by applying the method for evaluating the interfacial shear strength via a short-beam shear test proposed by Quan et al. [23,24]. Using this method, the stress concentration factor derived from the phase structure of the molded product can be quantified in the triaxial direction. In addition, since this method generates only shear stress, the yield condition can be limited to shear yield. When mechanical evaluation is performed via the tensile testing of polymer blend molded products, the two yield conditions are interface delamination and shear yield, and it is difficult to identify the factor of stress at yield initiation, but when combined with the results of short-beam shear testing, the yield condition due to tensile loading of the polymer blend can be identified. Based on the discussion presented above, the primary objective of this study of polymer blend injection-molded parts is using short-beam shear testing for the evaluation of mechanical anisotropy as a stress concentration factor, and clarifying the correlation between the obtained values and the phase structure. Furthermore, because only shear yielding occurs in short-beam shear testing, the yielding conditions attributable to uniaxial tensile loading were identified by comparing the results with those obtained from three-point bending testing. Because multiple phase structures were obtained in this study, the stress at shear yield initiation obtained for each phase structure was also modeled to clarify the correlation between the phase structure and the stress at yield initiation.

## 2. Materials and Methods

### 2.1. Materials

Table 1 presents the materials used for this study. The table also shows the melt flow rate (MFR), an index of melt viscosity. Two types of polypropylene were used: homo-type polypropylene (H-PP) and block-type polypropylene (B-PP). The block-type PP used in this paper is a propylene-ethylene block copolymer containing 16 wt.% ethylene-propylene rubber with an average molecular weight of 450,000 g/mol [25]. Styrene–ethylene–butadiene–styrene (SEBS) copolymers of two types were used to control the phase structure: SEBS with a low styrene ratio was designated as L-SEBS, and SEBS with a high styrene ratio was designated as H-SEBS.

### 2.2. Sample Preparation

**Figure 1:**
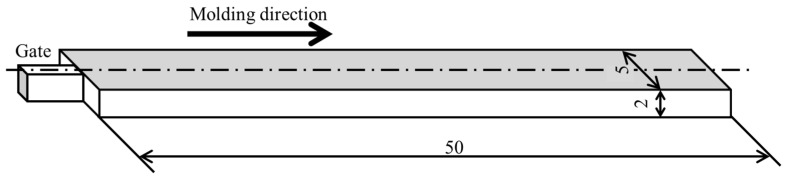
Beam specimen geometry (unit: mm) [24].

After these materials were poured into a twin-screw extruder (IMC-00 type, L/D = 25; Imoto Machinery Co., Ltd., Kyoto, Japan) with a screw diameter of 15 mm, they were melt-kneaded. Table 2 presents the studied compositions. SEBS was added at the ratio shown in that table to promote a fine dispersion of the dispersed phase. For this study, the melt-kneading temperature was set as 230 °C. The screw speed was fixed as 60 rpm. The resulting strands were cut into granular pieces using a pelletizer; they were used as pellets. The resulting pellets were filled into an ultra-compact electric injection molding machine (C. Mobile 0813; Shinko Sellbic Co., Ltd., Tokyo, Japan) and were injection molded to obtain strip-shaped molded products with the dimensions presented in Figure 1. Table 3 shows the injection molding conditions. The molding adopted the same geometry as in the literature [24]. All temperatures and times for injection molding were fixed. The holding pressure was varied to provide a good molded product.

### 2.3. Mechanical Anisotropy Determination via Short-Beam Shear Testing [24]

**Figure 2:**
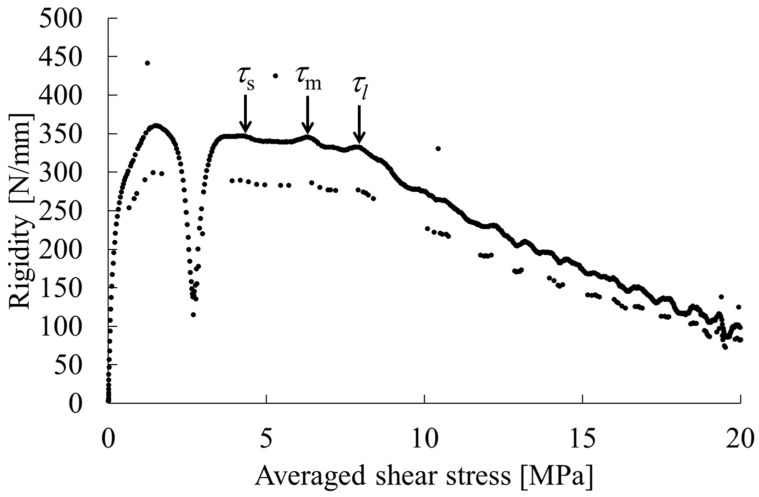
Rigidity—averaged shear stress curve obtained from short-beam shear tests [24].

Short-beam shear tests were conducted on a compact universal mechanical testing machine (MCT-2150; A&D Co., Ltd., Tokyo, Japan) using strips of molded products obtained via injection molding. The distance between spans was 10 mm. The loading speed was 10 mm/min. The obtained load–deflection curve is differentiated by the deflection to obtain the stiffness. The average shear stress *τ* was obtained from the load at each time point based on Equation (1).

(1) $$\tau =\frac{3P}{4A}$$

where *P* represents the load; and *A* stands for the cross-sectional area of the specimen. The stiffness-averaged shear stress curve was finally obtained using the calculations shown above. An example of a stiffness-averaged shear stress curve obtained from this test is presented in Figure 2. All of the materials examined for this study exhibited curves similar to this example. They show a rapid increase in stiffness at the beginning of loading followed by a discontinuous decrease. Subsequently, the stiffness becomes stable, but upon closer inspection, several points are apparent at which the stiffness decreases. As described in this paper, shear yielding is regarded as initiated at these points. Because shear stress is conjugate, when only shear stress occurs, this stress acts equally in the triaxial direction. Therefore, there would be, at most, three points of stiffness reduction. These points are *τ*_s_, *τ*_m_, and *τ*_l_, starting from the lowest value, where *τ*_s_ signifies the shear stress acting in the specimen thickness direction, *τ*_m_ in the specimen width direction, and *τ*_l_ in the flow direction. Using these values, mechanical anisotropy was determined as the minimum mechanical anisotropy *A*_s_ shown in Equation (2), the intermediate mechanical anisotropy *A*_m_ shown in Equation (3), and the maximum mechanical anisotropy *A*_l_ shown in Equation (4).

(2) $$A_{s}=1-\frac{\tau_{m}}{\tau_{l}}$$

(3) $$A_{m}=1-\frac{\tau_{s}}{\tau_{m}}$$

(4) $$A_{l}=1-\frac{\tau_{s}}{\tau_{l}}$$

These variables take values from 0 to 1, with a maximum of 1. In addition, from the definition, *A*_l_ ≥ *A*_m_.

Using these values, the stress concentration factors were determined via Equations (5)–(7) as *γ*_s_, *γ*_m_, and *γ*_l_, from largest to smallest.

(5) $$\gamma_{s}=\frac{\tau_{y}}{\sqrt{3}\tau_{s}}=\frac{1}{\sqrt{3}}\sqrt{1+\frac{1}{{\left(1-A_{m}\right)}^{2}}+\frac{1}{{\left(1-A_{l}\right)}^{2}}}$$

(6) $$\gamma_{m}=\frac{1}{\sqrt{3}}\sqrt{1+\frac{1}{{\left(1-A_{s}\right)}^{2}}+{\left(1-A_{m}\right)}^{2}}$$

(7) $$\gamma_{l}=\frac{1}{\sqrt{3}}\sqrt{1+{\left(1-A_{s}\right)}^{2}+{\left(1-A_{l}\right)}^{2}}$$

Their minimum value is 1 in the isotropic case. Their maximum value is theoretically infinite. Furthermore, using the stress concentration factor obtained from Equation (5) and *τ*_s_, the stress at yield initiation in the MD direction was obtained using Equation (8).

(8) $$\sigma_{y,MD}=3\gamma_{s}\tau_{s}$$

The stress at yield initiation obtained here is the value obtained at shear yielding. As described in this paper, the correlation between these stress concentration factors, the stress at yield initiation, and the phase structure is investigated.

### 2.4. Morphology Observation

**Figure 3:**
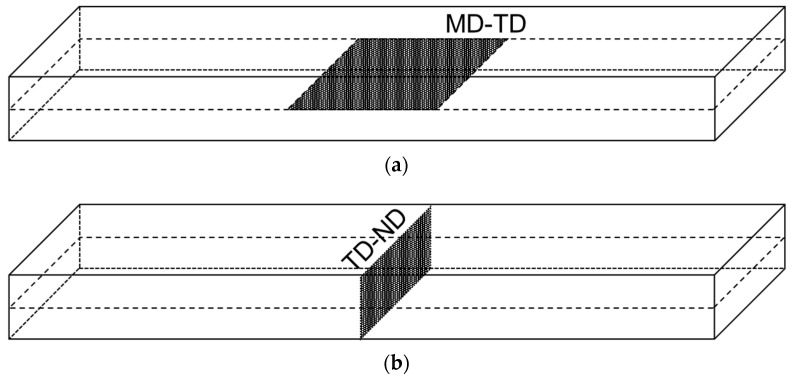
Morphology observation area. (**a**) MD-TD plane. (**b**) TD-ND plane.

Sections were prepared from the injection-molded specimen using a microtome (RX-860; Yamato Kohki Industrial Co., Ltd., Asaka, Japan). The phase structure was observed using a phase contrast microscope (BA410EPH-1080; Shimadzu Rika Corp., Tokyo, Japan). Compositions containing a substantial amount of PS were examined using scanning electron microscopy (SEM, Technex Co., Ltd., Tokyo, Japan, Tiny-SEM 510) on the cut surfaces created during section preparation to confirm the phase structure due to the difficulty in confirming it via phase contrast observation. Figure 3 shows the locations used for observation of the phase structure. We attempted to observe the phase structure three-dimensionally by observing the area corresponding to the core layer of the molded product from two angles: the MD-TD plane and the TD-ND plane. From the MD-TD plane image, 100 dispersed phases were extracted from the phase difference image. The aspect ratio was calculated as the ratio of lengths in the MD and TD directions. We attempted to achieve the purposes of this study by clarifying the degrees of correlation between the obtained phase structures and aspect ratios and the stress concentration factors in each direction.

## 3. Results and Discussions

### 3.1. Relation of Morphology and Mechanical Anisotropy of Injection-Molded PP-Rich Polymer Blends

**Figure 4:**
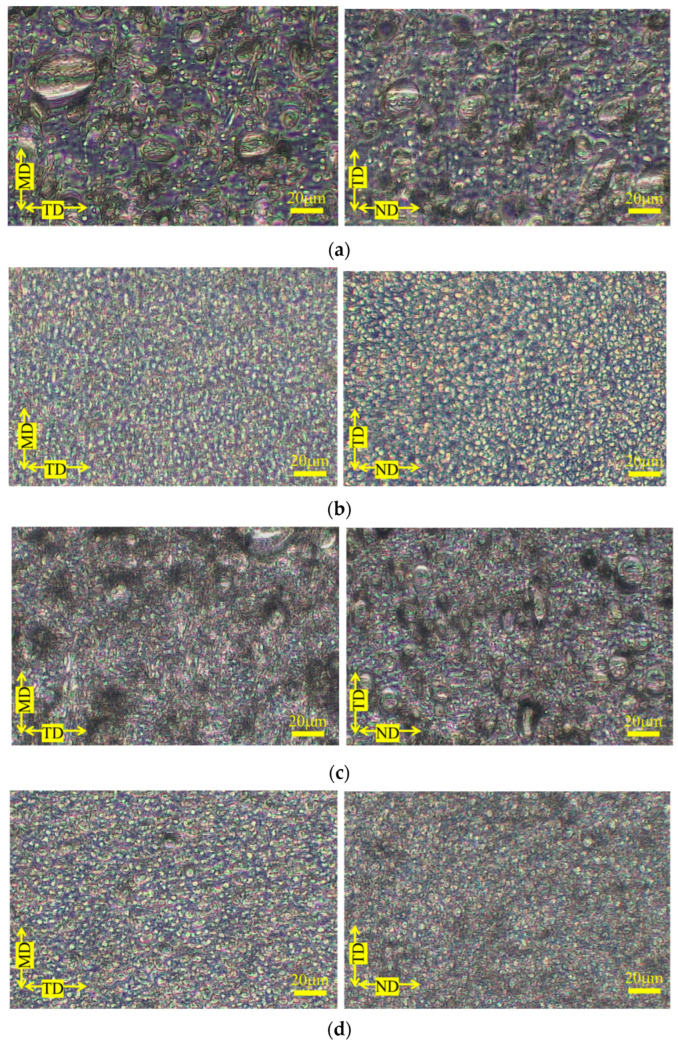
Phase contrast microscopic observations of the composition with high PP content. (**a**) H-PP/PS = 77.0/23.0 vol.%. (**b**) H-PP/PS/SEBS = 69.2/25.2/5.6 vol.%. (**c**) B-PP/PS = 77.0/23.0 vol.%. (**d**) B-PP/PS/SEBS = 69.2/25.2/5.6 vol.%.

**Figure 5:**
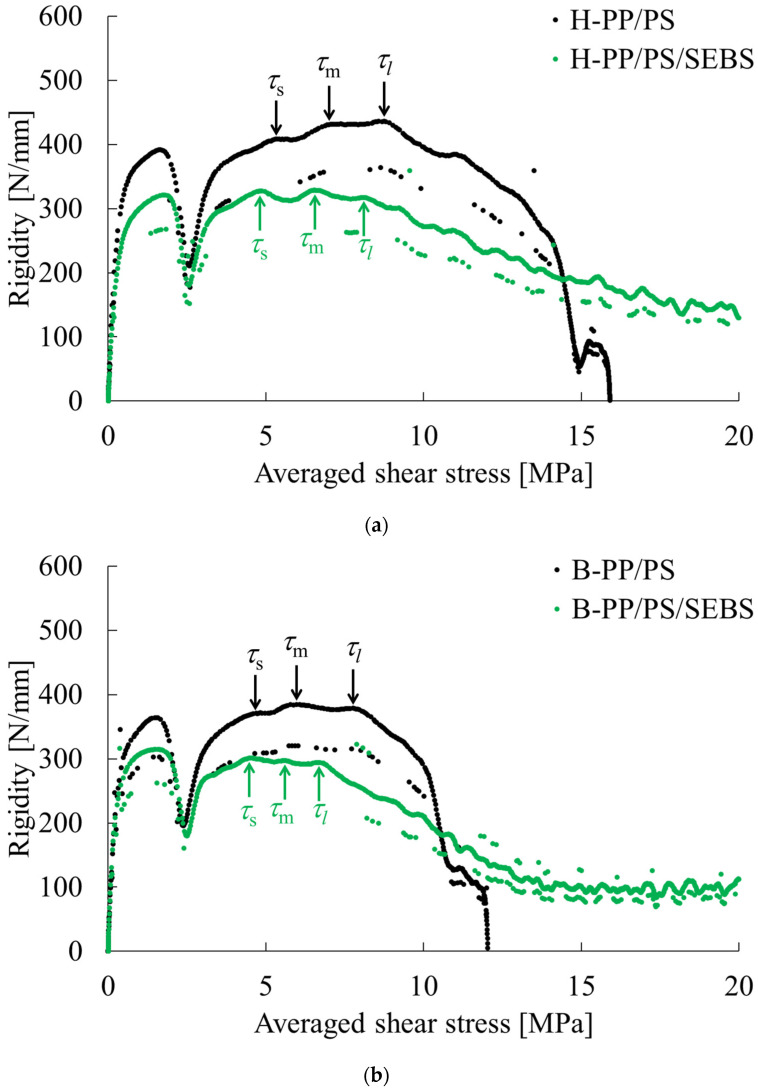
Rigidity—averaged shear stress curves of PP-rich polymer blends obtained via short-beam shear testing. (**a**) H-PP/PS = 77.0/23.0 vol.% and H-PP/PS/SEBS = 69.2/25.2/5.6 vol.%. (**b**) B-PP/PS = 77.0/23.0 vol.% and B-PP/PS/SEBS = 69.2/25.2/5.6 vol.%.

Figure 4 shows the phase contrast microscopy results for the PP-rich compositions; both MD-TD and TD-ND cross sections of the PP-rich compositions show a sea-island structure [7,8,9], leading to the conclusion that the phase structure in the PP-rich compositions is a sea-island structure. The addition of SEBS to this composition also resulted in a sea-island structure; the size of the dispersed phase appeared to be smaller and the particle size distribution narrower than that of the product without SEBS. Figure 5 portrays examples of stiffness-averaged shear stress curves for compositions with high PP content. The addition of SEBS tended to shift the shear stress at the onset of the yield in each direction toward the low stress side. Table 4 shows the shear stress at yield, mechanical illegality and stress concentration factors in each direction obtained via short-beam shear tests for the PP-rich compositions. This indicates that the morphology of the dispersed phase of the PP-rich composition is almost unchanged depending on the type of PP and the presence or absence of SEBS.

### 3.2. Relation of Morphology and Mechanical Anisotropy of Injection-Molded PS-Rich Polymer Blends

**Figure 6:**
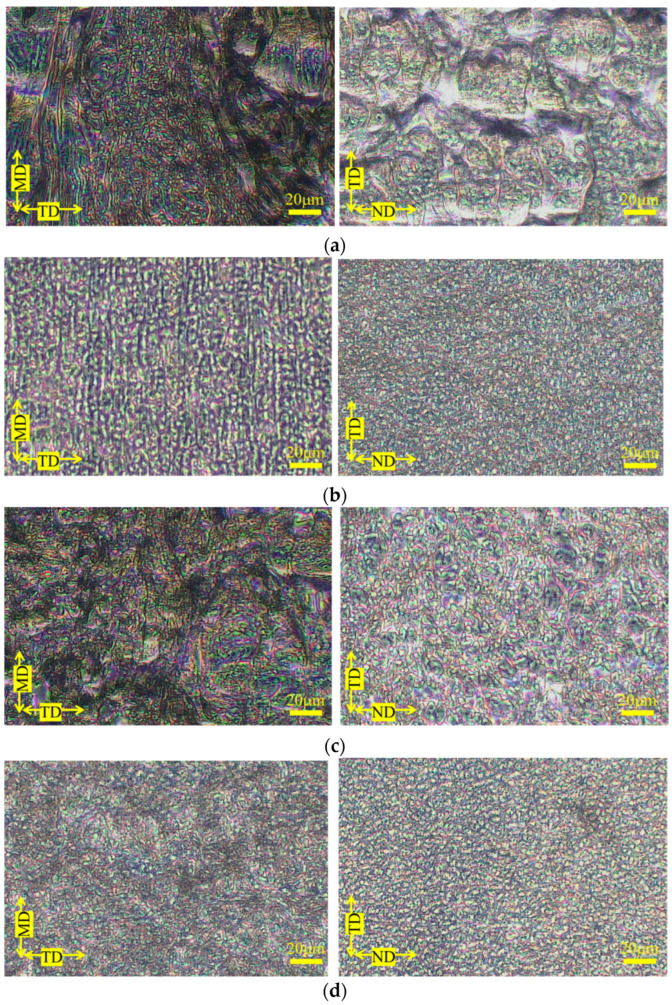
Phase contrast microscopic observations of the composition with high PS content. (**a**) H-PP/PS = 33.3/66.7 vol.%. (**b**) H-PP/PS/SEBS = 31.4/63.4/5.2 vol.%. (**c**) B-PP/PS = 33.3/66.7 vol.%. (**d**) B-PP/PS/SEBS = 31.4/63.4/5.2 vol.%.

**Figure 7:**
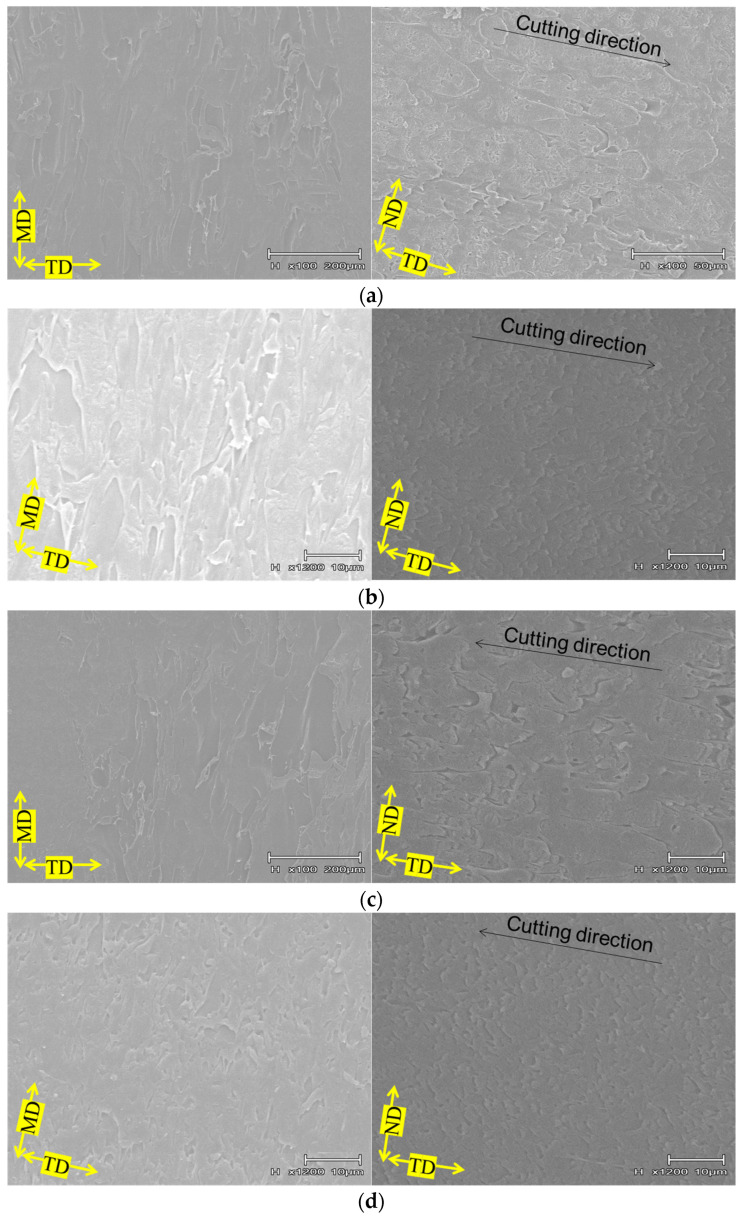
Scanning electron microscope observations of the composition with high PS content. (**a**) H-PP/PS = 33.3/66.7 vol.%. (**b**) H-PP/PS/SEBS = 31.4/63.4/5.2 vol.%. (**c**) B-PP/PS = 33.3/66.7 vol.%. (**d**) B-PP/PS/SEBS = 31.4/63.4/5.2 vol.%.

**Figure 8:**
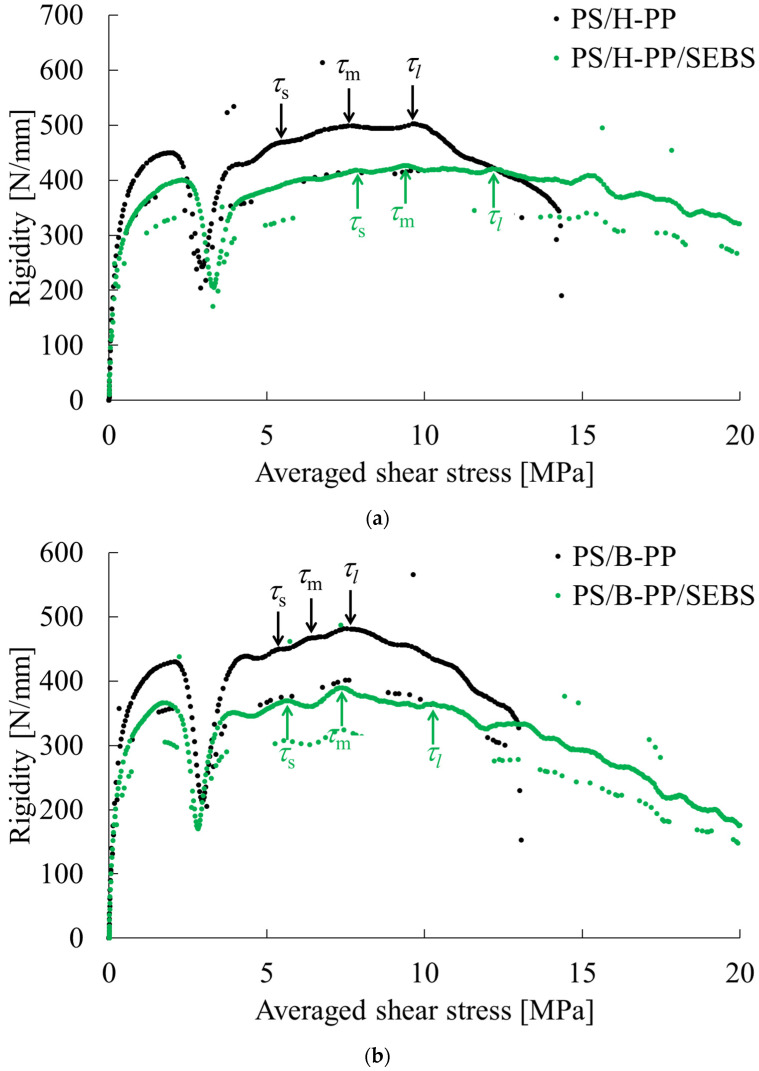
Rigidity—averaged shear stress curves of PS-rich polymer blends obtained via short-beam shear testing. (**a**) H-PP/PS = 33.3/66.7 vol.% and H-PP/PS/SEBS = 31.4/63.4/5.2 vol.%. (**b**) B-PP/PS = 33.3/66.7 vol.% and B-PP/PS/SEBS = 31.4/63.4/5.2 vol.%.

The phase contrast microscopy and scanning electron microscopy results of the PS-rich composition are shown in Figure 6 and Figure 7, respectively. For the SEBS-free variant, in the MD-TD cross section, both phase contrast and SEM images displayed an elliptical dispersed phase elongated towards the MD direction. The SEM image of TD-ND cross section also presented an elliptical dispersed phase elongated towards the TD direction. From these results, it was inferred that the dispersed phase in the PS-rich composition without SEBS is dispersed in an elongated disk shape. Upon the addition of SEBS to PS/H-PP, the phase contrast image exhibited a dispersed phase elongated in the MD direction, with a rod-like structure, while the TD-ND cross section showed a sea-island pattern. Furthermore, via the SEM image of the TD-ND cross section, it was revealed that the dispersed phase was finely dispersed. Thus, we can conclude that the addition of SEBS to PS/H-PP resulted in a phase morphology with a cylindrical dispersed phase with a relatively small diameter. Adding SEBS to PS/B-PP resulted in the dispersed phase arranging into a network, as observed in the phase contrast image of the MD-TD cross section. The TD-ND cross section’s phase contrast image and SEM image further confirmed a sea-island structure. These findings suggest that incorporating SEBS into PS/B-PP results in the dispersed phase forming a two-dimensional network structure. Figure 8 presents an example of stiffness-averaged shear stress curves for a composition with a high PS content. The addition of SEBS to PS/H-PP tended to shift the shear stress at the onset of yield in each direction toward the high stress side, whereas the addition of SEBS to PS/B-PP tended to shift the shear stress at the onset of yield in each direction toward the low stress side.

Table 5 presents the shear stress at yield initiation, mechanical anisotropy factors, and stress concentration factors in each direction obtained from short-beam shear tests for the PS-rich compositions. The stress concentration factors of the PS-rich compositions did not change with the type of PP when SEBS was not added; they were almost constant. When SEBS was added to these compositions, *γ*_s_ and *γ*_m_ tended to increase, although *γ*_l_ tended to decrease. This result suggests that the morphology of the dispersed phase is almost identical in the compositions with high PS content without SEBS, but that the morphology differs when SEBS is added.

**Figure 9:**
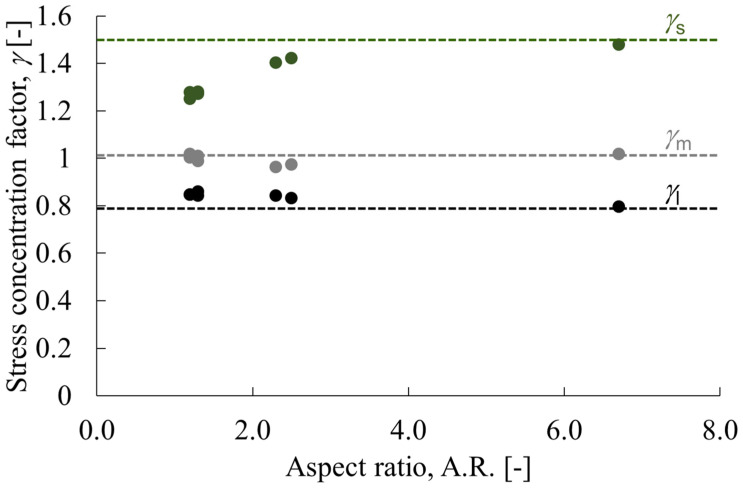
Relations between the aspect ratio and stress concentration factors. The figure displays the value when A.R. is infinite with a dashed line.

These results indicate that the mechanical anisotropy of PP/PS polymer blends depends on the morphology of the dispersed phase and indicate that it is only slightly affected by its size or its distribution. Based on these results, we investigated the degree of correlation between the aspect ratio, which is related to the morphology of the dispersed phase, and each stress concentration coefficient. Figure 9 shows the relation between the aspect ratio and each stress concentration coefficient obtained from phase contrast microscopic observations. Table 4b and Table 5b show the average values of the aspect ratios. The compositions which form a two-dimensional network are assumed to have continuity in the dispersed phase. The aspect ratio is assumed to be infinite, as represented by the dashed line in that figure. The *γ*_s_ and *γ*_m_ values tend to increase, although *γ*_l_ tends to decrease, as the aspect ratio increases. The *γ*_s_ and *γ*_l_ values change asymptotically toward the value of the aspect ratio at infinity. These results indicate that the mechanical anisotropy of PP/PS polymer blends is correlated with the aspect ratio of the dispersed phase. When comparing particle-dispersed and fiber-dispersed composites, it has been found that the latter display greater mechanical anisotropy. As the aspect ratio increases, these composites develop mechanical anisotropy comparable to that of continuous fibers [26,27,28]. This correlation is believed to be applicable to blends of PP and PS polymers.

### 3.3. Yield Conditions for PP/PS Polymer Blends

From the obtained values of mechanical anisotropy, the yield initiation stress in the MD direction was obtained using Equation (8). Table 6 presents the results obtained from comparing the yield initiation stress in the MD direction with the yield initiation stress *σ*_y,exp_ obtained from a three-point bending test under the same conditions as those reported in the literature [19]. *σ*_y,exp_ is expressed as in Equation (9).

(9) $$\sigma_{y,exp}=\frac{2\sigma_{f}}{3(1+\upsilon )}$$

In that equation, *σ*_f_ represents the flexural strength: *ν* is Poisson’s ratio. The value *ν* of the polymer blend is obtained using Equation (10).

(10) $$\upsilon =\sum_{i=1}^{n}\upsilon_{i}V_{i}$$

In that equation, *V* stands for the volume content; and *n* denotes the number of compositions. The fact that *σ*_y,MD_ is greater than *σ*_y,exp_ suggests that the yielding conditions generated by the short-beam shear test differ from those generated by the three-point bending test. Because the short-beam shear test imparts only shear stress, the only applicable yield condition is shear yielding. By contrast, the three-point bending test imparts mainly vertical stress, which results in expansion stress. Under these stress conditions, yielding might occur because of debonding at the interface in addition to shear yielding. Table 7 presents the yield conditions for the compositions examined for this study. To confirm this point, a model was constructed for this study to ascertain the yielding initiation stress when yielding occurs under the conditions of interface debonding.

When obtaining a molded product through the melt forming process, the product always undergoes a heating and cooling process. Therefore, thermal strain is expected to remain inside the molded product. For polymer blends, multiple phases with different coefficients of thermal expansion are mixed together. Therefore, thermal strain corresponding to the difference is assumed to occur at the interface. This strain is expressed as *ε*_v_ in Equation (11).

(11) $$\varepsilon_{v}=3\left(\alpha_{m}V_{m}-\sum_{j=1}^{n-1}\alpha_{j}V_{j}\right)\Delta T$$

where *α* is the coefficient of linear expansion. Also, Δ*T* expresses the difference between the molding process temperature and the test temperature. This strain is positive in the direction of contraction. When the expansion strain is generated by an external force and the strain reaches *ε*_v_, the interface will delaminate. In the case of a linear elastic body, if the necessary expansion stress at that time is *σ*_v_, it can be expressed as in Equation (12).

(12) $$\sigma_{v}=\varepsilon_{v}K$$

In that equation, *K* denotes the bulk modulus. Equation (13) is used to ascertain the bulk modulus *K*_p_ of the polymer blend.

(13) $$K_{p}=\sum_{i=1}^{n}K_{i}V_{i}$$

When the strain is small, the expansion stress produced by uniaxial tensile loading is 1/3 of the applied vertical stress. Therefore, the vertical stress *σ*_d_ required to produce an expansion strain of *ε*_v_ can be expressed as shown in Equation (14).

(14) $$\sigma_{d}=\frac{\sigma_{v}}{3}=\left(\alpha_{m}V_{m}-\sum_{j=1}^{n-1}\alpha_{j}V_{j}\right)\Delta TK$$

For this study, *σ*_d_ was obtained using the theory above. One-third of the difference from *σ*_y,exp_ was obtained as *σ*_i_. The values of *α*, *ν*, and *K* of each polymer were obtained using the method described in the literature [29]. Table 8 presents those results and density *ρ*. The volume fraction of each material was obtained using the *ρ* and weight ratio of each material. Table 9 presents the obtained volume fractions and *σ*_y,exp_, *σ*_v_ from Equation (12), and *σ*_d_ from Equation (14). In other words, if yielding occurs because of interface debonding, *σ*_y,exp_ is obtainable via Equation (15).

(15) $$\sigma_{y,exp}=\sigma_{d}+3\sigma_{i}$$

*σ*_i_ denoting a simple blend of PP and PS takes a negative value. Therefore, an interaction force is generated in the direction of interface separation. The addition of SEBS causes an increase in *σ*_i_, which means that the addition of SEBS generates a stronger force in the direction of interface debonding. This increase in force is thought to have promoted the micro-dispersion of the phase structure.

However, when comparing *σ*_y,exp_ and *σ*_y,MD_ for compositions with more PS, both values were almost identical, which indicates that the yield condition is shear yield. Although the yield initiation stress increased with the addition of SEBS, no change occurred in the yield condition. The reason for this lack of change is discussed by modeling the yield initiation stress initiated by shear yielding and by comparing it with the experimentally obtained results.

When the distributed phases are loaded in the direction of elongation, strain occurs equally in each phase. When the yield initiation strain of either phase is reached, the yield initiation of the entire system is regarded as occurring. In this case, the shear yield initiation stress of the polymer blend *σ*_yp_ is expressed as in Equation (16).

(16) $$\sigma_{yp}=3\sqrt{3}\left(\sum_{i=1}^{n}\alpha_{i}V_{i}\right)∆TK_{p}\left(1-2\upsilon \right)\cos\theta +\sqrt{3}\sigma_{i}\cos\theta =\sigma_{y,s}+\sqrt{3}\sigma_{i}\cos\theta$$

where *θ* represents the shear angle. The shear yield initiation stress obtained by excluding the *σ*_i_ component in Equation (16) above is presented in Table 10 as *σ*_y,s_. The calculated value of *σ*_y,s_ is greater than *σ*_y,MD_. This difference can be attributed to *σ*_i_. In this case, *σ*_i_ was obtained by transforming Equation (16) into Equation (17).

(17) $$\sigma_{i}=\frac{\sigma_{yp}-\sigma_{y,s}}{\sqrt{3}\cos\theta }$$

Table 10 also shows the value of *σ*_i_ obtained via Equation (17). The value of *σ*_i_ of the phase structure elongated in the loading direction is smaller than that found for the sea-island structure. The change caused by the addition of SEBS is slight. The reason for this slight change might be that the phase structure changes with the addition of SEBS into PS-rich compositions. Also, *σ*_i_ can be expressed as in Equation (18) using the interfacial interaction force and the specific surface area at the interface.

(18) $$\sigma_{i}=F_{i}S_{i}$$

In that equation, *F*_i_ stands for the interfacial interaction force; and *S*_i_ denotes the specific surface area of the interface. *S*_i_ depends on the volume content of the dispersed phase and its morphology. For example, for the same volume fraction and the same diameter, a 1.5:1 relation exists between the sizes of the specific surface area when dispersed in a spherical form and when dispersed in a rod form. In other words, even though the addition of SEBS acted in the direction of increasing *F*_i_, the change in *σ*_i_ was regarded as minute and as a result of the decrease in the specific surface area at the interface because of the change in phase structure. These results indicate that the shear yield initiation stress of a polymer blend with a dispersed phase elongated in the loading direction is obtainable from the shear yield initiation stress considering the interfacial interaction force.

The shear yield initiation stress of the PP-rich compositions is also discussed. Figure 6 and Figure 7 show that in these compositions, a sea-island structure is formed with and without SEBS. In this structure, the interfacial interaction forces are isotropic and might cancel each other out. Furthermore, because the dispersed phase is spherical, we inferred that the logarithmic complex law shown in Equation (19) is valid [30].

(19) $$\sigma_{y,p}=e^{\left\{\sum_{i=1}^{n}(V_{i}\ln\sigma_{yi})\right\}}$$

The interfacial interaction forces are not considered in *σ*_yi_ here. The shear yield initiation stress obtained using Equation (19) above is shown in Table 9 as *σ*_y,p_. The calculated *σ*_y,p_ shows good agreement with *σ*_y,MD_. These results indicate that the shear yield initiation stress of a polymer blend with a spherical dispersed phase is obtainable by averaging the shear yield initiation stress without considering the interfacial interaction force using the logarithmic compound law.

As described in this paper, short-beam shear tests were performed on PP/PS polymer blends to evaluate their mechanical anisotropy with respect to yield initiation stress. The results showed a correlation between the phase structure and the evaluated mechanical anisotropy, suggesting that there is anisotropy in fracture toughness and other mechanical properties related to yield initiation stress. To assess fracture toughness, an anisotropic evaluation of notched impact strength has been performed on 3D molded parts [31]. Additional studies will also be conducted for injection-molded products. Anisotropy associated with the fracture toughness of polymer blends is more pronounced than yield initiation stress. For industrial considerations, elucidating this mechanism is particularly important. The mechanisms of anisotropy affecting fracture toughness will be further clarified in future studies based on the findings presented here.

## 4. Conclusions

For this study, short-beam shear tests were performed on injection-molded PP/PS polymer blends to evaluate their anisotropy with respect to the shear yield initiation stress. The correlation between the obtained anisotropy and the phase structure inside the injection-molded products was investigated. The correlation between the phase structure and the shear yield initiation stress was modeled together with the yield conditions identified via a comparison with the three-point bending test results. The relevant findings are presented below.

- When PP is a continuous phase, the phase structure forms a sea-island structure. The yield condition under uniaxial tensile loading was interface debonding.
- When PS is a continuous phase, the phase structure has a dispersed phase that is elongated in the flow direction. This structure was changed by the addition of SEBS. The yielding condition under uniaxial tensile loading was shear yielding.
- The aspect ratio of the dispersed phase was correlated with the stress concentration factor.
- When the PP forming the sea-island structure is a continuous phase, the shear yield initiation stress is explainable by the log-complex law without considering the interfacial interaction stress.
- When the PS forming the structure with stretched dispersed phase is a continuous phase, the shear yield initiation stress is explainable by the shear yield initiation stress considering the interfacial interaction force.

## Figures and Tables

**Table 1:** Material information.

Material	Code	Manufacturer	Name	MFR (g/10 min)
PP	H-PP	Japan Polypropylene Corp., Tokyo, Japan	Novatec-PP MA1B	21@230 °C, 2.160 kgf
	B-PP	Japan Polypropylene Corp., Tokyo, Japan	Novatec-PP BC03B	30@230 °C, 2.160 kgf
PS	PS	Toyo Styrene Co., Ltd., Tokyo, Japan	Toyo styrene G210C	10@230 °C, 2.160 kgf
SEBS	L-SEBS	Asahi Kasei Corp., Tokyo, Japan	Tuftec H1052	13@230 °C, 2.160 kgf
	H-SEBS	Asahi Kasei Corp., Tokyo, Japan	Tuftec H1043	2@230 °C, 2.160 kgf

**Table 2:** Material compositions.

H-PP (vol.%)	B-PP (vol.%)	PS (vol.%)	L-SEBS (vol.%)	H-SEBS (vol.%)
100.0				
77.0		23.0		
69.2		25.2	4.0	1.6
33.3		66.7		
31.4		63.4	1.7	3.5
		100.0		
	100.0			
	77.0	23.0		
	69.2	25.2	4.0	1.6
	33.3	66.7		
	31.4	63.4	1.7	3.5
		100.0		

**Table 3:** Injection molding conditions.

H-PP (vol.%)	B-PP (vol.%)	PS (vol.%)	L-SEBS (vol.%)	H-SEBS (vol.%)	*T*_inj_ (°C)	*T*_mold_ (°C)	*V*_inj_ (mm/s)	*P*_hold_ (MPa)	*T*_inj_ (s)	*T*_cool_ (s)
100					230	50	30	46	10	15
77.0		23.0			230	50	30	46	10	15
69.2		25.2	4.0	1.6	230	50	30	49	10	15
33.3		66.7			230	50	30	56	10	15
31.4		63.4	1.7	3.5	230	50	30	56	10	15
		100.0			230	50	30	46	10	15
	100.0				225	50	30	46	10	15
	77.0	23.0			230	50	30	46	10	15
	69.2	25.2	4.0	1.6	230	50	30	49	10	15
	33.3	66.7			230	50	30	56	10	15
	31.4	63.4	1.7	3.5	230	50	30	56	10	15
		100.0			230	50	30	46	10	15

**Table 4:** Mechanical anisotropy of PP/PS polymer blends with high PP content.

**(a) Mechanical Anisotropy Factor**										
**H-PP** **(vol.%)**	**B-PP** **(vol.%)**	**PS** **(vol.%)**	**L-SEBS** **(vol.%)**	**H-SEBS** **(vol.%)**	***τ*_s_** **(MPa)**	***τ*_m_** **(MPa)**	***τ*_l_** **(MPa)**	***A*_s_** **(-)**	***A*_m_** **(-)**	***A*_l_** **(-)**
100					4.2	6.1	8.1	0.25	0.31	0.48
77.0		23.0			5.5	7.0	8.3	0.16	0.21	0.34
69.2		25.2	4.0	1.6	5.2	6.7	7.7	0.13	0.22	0.32
	100				4.5	5.6	6.6	0.15	0.20	0.32
	77.0	23.0			5.2	6.6	7.9	0.16	0.21	0.34
	69.2	25.2	1.7	3.5	4.4	5.4	6.5	0.17	0.19	0.32
		100.0			13.1	16.2	18.8	0.14	0.19	0.30
**(b) Stress concentration factor and aspect ratio**										
**H-PP** **(vol.%)**	**B-PP** **(vol.%)**	**PS** **(vol.%)**	**L-SEBS** **(vol.%)**	**H-SEBS** **(vol.%)**	***γ*_s_** **(-)**		***γ*_m_** **(-)**	***γ*_l_** **(-)**		**A.R.** **(-)**
100					1.51		1.04	0.78		-
77.0		23.0			1.28		1.00	0.85		1.2
69.2		25.2	4.0	1.6	1.27		0.99	0.86		1.3
	100				1.25		1.01	0.85		-
	77.0	23.0			1.28		1.01	0.84		1.3
	69.2	25.2	4.0	1.6	1.25		1.02	0.85		1.2
		100.0			1.24		1.00	0.86		-

**Table 5:** Mechanical anisotropy of PP/PS polymer blends with high PS content.

**(a) Mechanical Anisotropy Factor**										
**H-PP** **(vol.%)**	**B-PP** **(vol.%)**	**PS** **(vol.%)**	**L-SEBS** **(vol.%)**	**H-SEBS** **(vol.%**)	***τ*_s_** **(MPa)**	***τ*_m_** **(MPa)**	***τ*_l_** **(MPa)**	***A*_s_** **(-)**	***A*_m_** **(-)**	***A*_l_** **(-)**
100.0					4.5	5.6	6.6	0.15	0.20	0.32
33.3		66.7			5.2	7.6	8.9	0.15	0.32	0.42
31.4		63.4	1.7	3.5	6.4	9.3	11.9	0.22	0.31	0.46
	100.0				4.5	5.6	6.6	0.15	0.20	0.32
	33.3	66.7			4.8	7.0	8.0	0.13	0.31	0.40
	31.4	63.4	1.7	3.5	5.6	8.2	10.5	0.22	0.32	0.47
		100.0			13.1	16.2	18.8	0.14	0.19	0.30
**(b) Stress concentration factor and aspect ratio**										
**H-PP** **(vol.%)**	**B-PP** **(vol.%)**	**PS** **(vol.%)**	**L-SEBS** **(vol.%)**	**H-SEBS** **(vol.%)**	***γ*_s_** **(-)**	***γ*_m_** **(-)**	***γ*_l_** **(-)**	**A.R.** **(-)**		
100.0					1.25	1.01	0.85	-		
33.3		66.7			1.42	0.97	0.83	2.5		
31.4		63.4	1.7	3.5	1.48	1.02	0.80	6.7		
	100.0				1.25	1.01	0.85	-		
	33.3	66.7			1.40	0.96	0.84	2.3		
	31.4	63.4	1.7	3.5	1.49	1.02	0.79	∞		
		100.0			1.24	1.00	0.86	-		

**Table 6:** Results of comparing the yield initiation stress in the MD direction with the yield initiation stress *σ*_y,exp_ obtained from a three-point bending test.

H-PP (vol.%)	B-PP (vol.%)	PS (vol.%)	L-SEBS (vol.%)	H-SEBS (vol.%)	*τ*_s_ (MPa)	*γ*_s_ (-)	*σ*_y,MD_ (MPa)	*σ*_fy,exp_ (MPa)	*υ* (-)	*σ*_y,exp_ (MPa)
100					4.2	1.51	19.0	28.0	0.407	19.9
77.0		23.0			5.5	1.28	21.1	25.5	0.392	18.3
69.2		25.2	4.0	1.6	5.2	1.27	19.8	23.6	0.393	16.9
33.3		66.7			5.2	1.42	22.2	31.5	0.363	23.1
31.4		63.4	1.7	3.5	6.4	1.48	28.4	38.0	0.366	27.8
	100.0				4.5	1.25	16.9	24.1	0.413	17.1
	77.0	23.0			5.2	1.28	20.0	22.2	0.398	15.9
	69.2	25.2	4.0	1.6	4.4	1.25	16.5	19.9	0.398	14.3
	33.3	66.7			4.8	1.40	20.2	28.0	0.366	20.5
	31.4	63.4	1.7	3.5	5.6	1.49	25.0	32.2	0.368	23.5
		100.0			13.1	1.24	48.6	65.5	0.342	48.8

**Table 7:** Yield conditions for the compositions.

H-PP (vol.%)	B-PP (vol.%)	PS (vol.%)	L-SEBS (vol.%)	H-SEBS (vol.%)	*σ*_y,MD_ (MPa)	*σ*_y,exp_ (MPa)	*YC*_MD_
77.0		23.0			21.1	18.3	Debonding
69.2		25.2	4.0	1.6	19.8	16.9	Debonding
33.3		66.7			22.2	23.1	Shear yield
31.4		63.4	1.7	3.5	28.4	27.8	Shear yield
	77.0	23.0			20.0	15.9	Debonding
	69.2	25.2	4.0	1.6	16.5	14.3	Debonding
	33.3	66.7			20.2	20.5	Shear yield
	31.4	63.4	1.7	3.5	25.0	23.5	Shear yield

**Table 8:** Physical properties of polymers.

	FS (MPa)	FM (MPa)	*α* (10^−5^/K)	*υ* (-)	*E* (MPa)	*K* (MPa)	*ρ* (g/cm^3^)
H-PP	42.1	1728	9	0.407	767	1374	0.9
B-PP	36.3	1469	10	0.412	623	1181	0.9
PS	98.2	3237	7	0.342	2100	2215	1.04
L-SEBS	1.6	40	61	0.481	4	38	0.89
H-SEBS	46	1200	11	0.367	693	868	0.97

**Table 9:** Interfacial interaction stress for compositions yielding caused by interfacial debonding.

H-PP (vol.%)	B-PP (vol.%)	PS (vol.%)	L-SEBS (vol.%)	H-SEBS (vol.%)	*YC*_MD_	*σ*_y,exp_ (MPa)	*K* (MPa)	*σ*_d_ (MPa)	*σ*_i_ (MPa)
77.0		23.0			Debonding	18.3	1567	163.8	−48.5
69.2		25.2	4.0	1.6	Debonding	16.9	1524	198.9	−60.6
	77.0	23.0			Debonding	15.9	1419	164.3	−49.5
	69.2	25.2	4.0	1.6	Debonding	14.3	1375	193.4	−59.7

**Table 10:** Experimentally obtained and calculated shear yield initiation stress.

H-PP (vol.%)	B-PP (vol.%)	PS (vol.%)	L-SEBS (vol.%)	H-SEBS (vol.%)	*σ*_y,MD_ (MPa)	*σ*_y,p_ or *σ*_y,s_ (MPa)	*σ*_i_ (MPa)	Structure
77.0		23.0			21.1	23.6	−48.5	Sea-island
69.2		25.2	4.0	1.6	19.8	21.0	−60.6	Sea-island
33.3		66.7			22.2	36.4	−9.5	Elongated Disc
31.4		63.4	1.7	3.5	28.4	38.0	−7.3	Cylinder
	77.0	23.0			20.0	21.4	−49.5	Sea-island
	69.2	25.2	4.0	1.6	16.5	19.2	−59.7	Sea-island
	33.3	66.7			20.2	34.5	−10.0	Elongated Disc
	31.4	63.4	1.7	3.5	25.0	36.1	−9.0	2D network

## Data Availability

The data presented in this study are available on request from the corresponding author.
